# Development, implementation, and evaluation of a train-the-trainer program for emerging nurse leaders in China: a 12-year longitudinal study

**DOI:** 10.1186/s12909-025-08280-7

**Published:** 2025-11-26

**Authors:** Jing Chen, Guowen Zhang, Agnes Tiwari, Sophia Siu Chee Chan

**Affiliations:** 1 The Jockey Club School of Public Health and Primary Care, Chinese University of Hong Kong, Hong Kong SAR, China; 2 School of Nursing, The University of Hong Kong, Hong Kong SAR, China; 3 School of Nursing, Hong Kong Sanatorium and Hospital, Hong Kong SAR, China; 4 School of Public Health, The University of Hong Kong, Hong Kong SAR, China

**Keywords:** Train-the-Trainer, Nursing leadership, Healthcare reform, Tobacco control, Smoking cessation

## Abstract

**Background:**

Despite being the largest group of health care professionals, nurses represent a neglected human resource in China, especially in leadership, education, and public health. The aim of this study was to develop and evaluate a Train-the-Trainer (TTT) model to build leadership capacity among emerging nurse leaders in South China, using a comprehensive assessment and evaluation immediately after, at 6-month, and 12-year post-TTT.

**Methods:**

A mixed-methods approach was employed starting with a needs assessment to guide the development and implementation of the curriculum in the TTT programs. Three tailored courses were developed. Outcomes were assessed using pre- and post-training surveys on participants’ knowledge, attitudes, practices (KAP), along with self-reported capability improvements. Multivariate analysis of variance (MANOVA) and paired t-tests were employed to assess the TTT impact. Cascading effects were assessed through reports of TTT activities and institutional changes. A *p*-value < 0.05 was considered as statistically significant.

**Results:**

A total of 180 emerging nurse leaders (middle career) from 136 institutions in Guangdong (GD) Province participated in the TTT program, with support from the Guangdong Province Department of Health. The course participants showed significant short-term improvement in KAP and self-reported competencies in the three courses, with sustained gains observed at 6 months and 12 years post-training (all *p* < 0.05). The cascading effect of the program was substantial at both 6-month and 12-year post-TTT, with 180 participants disseminating knowledge and delivering further training to about 25,000 healthcare professionals in the Province.

**Conclusions:**

The TTT program proved to be a scalable, sustainable model for developing nurse leadership and strengthening system capacity in China. In the global shortage of nurses, more than ever, we need to prepare emerging leaders to advance nursing practice and build a quality nursing workforce contributing to China’s national health reform.

## Background

Non-communicable diseases (NCDs), including cardiovascular diseases, cancer, and chronic respiratory diseases, have emerged as the leading causes of morbidity and mortality worldwide, disproportionately affecting low- and middle-income countries [1]. This growing burden of NCDs is driven by modifiable risk factors such as tobacco use, unhealthy diets, sedentary lifestyles, and harmful alcohol consumption. As a result, strengthening primary health care systems and building workforce capacity to address NCDs has become a global priority.

Nurses have a key role to play in the health care system in providing, expanding, connecting, and coordinating care, leveraging their deep understanding of patient, family, and system priorities. As the largest segment of the workforce, nurses are central to efforts aimed at achieving universal health coverage. In particular, nurses have been increasingly capacitated and engaged in the prevention and management of NCDs, through targeted training programs, task-shifting initiatives, and collaborative care models that enhance health promotion, early detection, treatment adherence, and long-term disease management [2–6]. Their growing involvement in linking primary care and public health is critical in addressing the multifactorial nature of NCDs [7]. The COVID-19 pandemic has further highlighted nurses’ resilience, self-care ability, and adaptability in the face of health system disruptions, reinforcing their indispensable role in current and future health challenges, including the fight against NCDs [8]. Beyond clinical care, nurses are emerging as leaders in public health and care coordination, essential for strengthening the health system [9]. With the global ageing population, rising health care challenges, and shortage of nurses, there is an urgent need to develop a critical mass of nurse leaders capable of advancing both care deliver and workforce quality to achieve health for all [9, 10], especially in settings where their roles have traditionally been underutilized.

Contemporary models of professional learning emphasize active, collaborative, and sustained development that is contextually grounded and closely aligned with professional goals. Key features include content relevance, active participation, coherence with learners’ needs, collective learning, and sufficient duration [11, 12]. These models represent a shift from traditional, one-off workshops to embedded, iterative learning experiences that lead to tangible improvements in professional practice and outcomes [13]. In clinical and healthcare settings, professional learning is largely experiential and workplace-based. It involves peer collaboration and ongoing reflection. Such learning occurs within communities of practice and remains closely tied to patient care and institutional objectives [14]. Building on these theoretical frameworks, one widely adopted model for building workforce capacity is the Train-the-Trainer (TTT) approach. Train-the-Trainer is an educational model to engage master trainers in coaching new trainers that are less experienced with a particular topic or skill, or with training overall. Successful applications in HIV care (Indonesia) [15], maternal health (UK) [16], and tobacco cessation training [17] demonstrate the model’s scalability and effectiveness. In nursing education, TTT models have been employed to promote clinical competence, enhance leadership, and ensure the sustainability of professional development across healthcare systems.

China’s rapid epidemiological and demographic transition, marked by rising NCD prevalence and an ageing population [18], prompted the government’s “Healthy China 2030” initiative to improve access, quality, and integration of care through its healthcare reform [19–21]. Strengthening the primary care workforce, particularly nurses, the largest segment of the health workforce, is essential. Yet nurses in China remain underutilized due to longstanding cultural norms, hierarchical healthcare structures, and limited policy support for advanced nursing roles [22, 23]. To address this, the School of Nursing of the University of Hong Kong (HKU), in collaboration with the Department of Health of Guangdong Province, launched the “Training-of-Trainers Programme for Advancement in Nursing” program (TTT program). This paper discusses the development, implementation, and evaluation of the TTT program, which aimed to equip a cohort of nurse trainers with advanced knowledge and leadership skills to train and mentor other nurses in Guangdong Province.

## Methods

### Study design, aim, and objectives

This study employed mixed-methods to assess the implementation and outcomes of the TTT program. The evaluation combined quantitative and qualitative approaches to capture changes in participants’ competencies, leadership development, and the broader impact on nursing practice. Data were collected through pre- and post-training assessments, participant feedback, and follow-up interviews over 12 years to evaluate both learning outcomes and knowledge transfer within healthcare institutions.

The primary aim of the TTT program was to build a cohort of nurse trainers with advanced knowledge and leadership skills to train and mentor other nurses in Guangdong Province. Its objectives were to: (1) enhance pedagogical skills of nurse educators; (2) strengthen management skills of nurse managers; (3) promote community nursing, particularly in chronic disease prevention and tobacco dependency treatment; and (4) establish a sustainable network of nurse trainers to localize knowledge, support peers, and drive ongoing initiatives. This cascading training approach was designed to strengthen nursing competencies at scale and improve healthcare quality in China.

### Setting and context of the study

This study was conducted in the context of China’s national “Healthy China 2030” policy, which emphasizes the development of a high-quality, accessible healthcare system capable of meeting the challenges posed by an ageing population and a growing burden of NCDs. As part of this initiative, efforts have been made to strengthen the primary care workforce through targeted training and capacity-building strategies. In line with these national priorities, the TTT program was developed to advance the professional competencies of nurse educators and leaders across Guangdong Province and to cultivate a sustainable network of nurse trainers capable of cascading knowledge and best practices throughout the healthcare system.

The initiative was jointly implemented by the University of Hong Kong, the Guangdong Department of Health, and Guangdong Nurse Education Centre (GNEC). The HKU team collaborated closely with provincial partners in program design, monitoring, and evaluation, while the GNEC served as the primary implementation partner, coordinating participant recruitment, managing logistics, and co-facilitating training activities in Guangzhou.

### Study population

Eligible participants were mid-career nurses with demonstrated leadership potential and a minimum number of years of professional experience in their respective roles. Participants were purposively sampled from 136 healthcare institutions across all 23 cities in Guangdong Province to ensure wide representation. Recruitment was guided by the above identified training needs from the preliminary assessment that corresponded to the three courses, i.e. Nursing Management course, Tobacco Dependency Nursing Intervention and Management course, and Nursing Education Course. Given the limited tobacco control training among nurses in China, this group was chosen to build capacity for smoking cessation intervention in both clinical and community settings. A total of 180 emerging nurse leaders participated in the TTT program (Table 1), with at least one to two mid-career nurses recruited from each of the 23 cities, representing major health care institutions in the province. In order to maximize the impact of the TTT program, a total of three identical Nursing Management courses, two identical Tobacco Dependency Nursing Intervention and Management courses, and one Nursing Education course were delivered.

**Table 1:** Characteristics of the participants in the TTT program

	Management	Tobacco	Education	Total
Number of participants	*N* = 90	*N* = 59*	*N* = 30	*N* = 179
	n (%)	n (%)	n (%)	n (%)
Gender				
Male	0 (0.0)	15 (25.4)	2 (6.7)	17 (9.5)
Female	90 (100.0)	44 (74.6)	28 (93.3)	162 (90.5)
Age				
26–30	5 (5.6)	17 (28.8)	6 (20.0)	28 (15.6)
31–35	26 (28.9)	14 (23.7)	6 (20.0)	46 (25.7)
36–40	46 (51.1)	9 (15.3)	11 (36.7)	66 (36.9)
41–45	10 (11.1)	5 (8.5)	4 (13.3)	19 (10.6)
46–50	3 (3.3)	11 (18.6)	3 (10.0)	17 (9.5)
51–55	0 (0.0)	3 (5.1)	0 (0.0)	3 (1.7)
Educational background				
Below technical secondary school level	1 (1.1)	1 (1.7)	0 (0.0)	2 (1.1)
Technical secondary school level	0 (0.0)	4 (6.8)	0 (0.0)	4 (2.2)
Junior college	13 (14.4)	11 (18.6)	0 (0.0)	24 (13.4)
Bachelor	74 (82.2)	36 (61.0)	14 (46.7)	124 (69.3)
Master/PhD	2 (2.2)	7 (11.9)	16 (53.3)	25 (14.0)
No. of years qualified as a registered nurse/public health practitioner				
1–5	1 (1.1)	7 (11.9)	5 (16.7)	13 (7.3)
6–10	8 (8.9)	13 (22.0)	4 (13.3)	25 (14.0)
11–15	17 (18.9)	9 (15.3)	5 (16.7)	31 (17.3)
16–20	43 (47.8)	10 (16.9)	5 (16.7)	58 (32.4)
> 20	21 (23.3)	16 (27.1)	6 (20.0)	43 (24.0)
Missing	0 (0.0)	4 (6.8)	5 (16.7)	9 (5.0)
No. of years worked in the present department/ward				
1–5	34 (37.8)	28 (47.5)	12 (40.0)	74 (41.3)
6–10	26 (28.9)	15 (25.4)	5 (16.7)	46 (25.7)
11–15	20 (22.2)	10 (16.9)	5 (16.7)	35 (19.6)
16–20	7 (7.8)	3 (5.1)	3 (10.0)	13 (7.3)
> 20	2 (2.2)	2 (3.4)	2 (6.7)	6 (3.4)
Missing	1 (1.1)	1 (1.7)	3 (10.0)	5 (2.8)
No. of years stayed in the current position				
1–5	74 (82.2)	36 (61.0)	22 (73.3)	132 (73.7)
6–10	16 (17.8)	17 (28.8)	7 (23.3)	40 (22.3)
11–15	0 (0.0)	3 (5.1)	1 (3.3)	4 (2.2)
16–20	0 (0.0)	2 (3.4)	0 (0.0)	2 (1.1)
Missing	0 (0.0)	1 (1.7)	0 (0.0)	1 (0.6)
No. of years of experience				
1–5	40 (44.4)	39 (66.1)	8 (26.7)	87 (48.6)
6–10	35 (38.9)	13 (22.0)	12 (40.0)	60 (33.5)
11–15	11 (12.2)	1 (1.7)	5 (16.7)	17 (9.5)
16–20	3 (3.3)	3 (5.1)	2 (6.7)	8 (4.5)
> 20	1 (1.1)	0 (0.0)	3 (10.0)	4 (2.2)
Missing	0 (0.0)	3 (5.1)	0 (0.0)	3 (1.7)
Previous training				
No	30 (33.3)	34 (57.6)	13 (43.3)	77 (43.0)
Yes	59 (65.6)	24 (40.7)	17 (56.7)	100 (55.9)
Missing	1 (1.1)	1 (1.7)	0 (0.0)	2 (1.1)

*Originally 60 participants attended the course. One did not finish the 2^nd^ part of the course in HK due to personal reason and was excluded from the final analysis.

For the three *Nurse Management courses*, a total of 90 participants were recruited from 81 institutions, and they were further divided into three subgroups with 30 participants for each of the three identical courses. They were all female and the majority of them were in their thirties. Over 70% had been qualified as a registered nurse for at least 10 years. About half of the participants had management experience of more than five years. They were mainly nurse managers in charge of a ward, while a few were the in-charge of a department.

For the *Tobacco Dependency Nursing Intervention and Management courses*, a total of 59 participants from 54 institutions completed the course, 27% of whom were male. 34 of them were nurses and 25 were public health physicians, the latter being the major healthcare providers in the community healthcare settings in China.

For the *Nursing Education course*, among the 30 participants who came from 18 institutions, two were male. The majority worked in School of Nursing at universities, and the rest were from the polytechnic and hospitals. The majority of them had been in a teaching post for more than five years, and half of the participants had previous training in education.

### Intervention design and development

The Training-of-Trainers (TTT) program was delivered as a 2-week intensive course, developed to strengthen nursing capacity in Guangdong Province. Training took place in both Guangzhou and Hong Kong. The design and development of the courses followed the ADDIE instructional design framework and is outlined below.

#### Analysis phase – needs assessment

Knowledge gaps and learning needs of nurse participants were identified through a mixed-methods needs assessment [24].

A comprehensive literature review was first conducted to identify global and regional priorities in nursing education and leadership building. Subsequently, qualitative data were collected to determine the unmet learning needs of local mid-career nurse leaders. Five focus groups and ten individual in-depth interviews were conducted in December 2009 with 38 senior and mid-level nurse managers from the participating hospitals in Guangzhou, China. Among participants, 86.8% were nurse managers, and over half had more than ten years of management experience. All interviews were tape-recorded, transcribed, and analysed using content analyses to identify common themes and training gaps. An initial coding framework was developed based on the interview guide and emergent themes, and then iteratively refined. Two independent researchers coded the transcripts separately using NVivo software, and discrepancies were resolved through discussion and consensus to ensure intercoder reliability. The analysis revealed strong interest in training relevant to daily practice, particularly communication, team building, quality assurance, and crisis management, and highlighted the need for institutional support to sustain training outcomes.

#### Design phase – curriculum framework

An Expert Committee was established, comprising three internationally renowned nurse experts, two leading local nurse experts, and one representative from the Guangdong Province Department of Health. Drawing upon the literature gaps and the findings of the above needs assessment, the Committee collaboratively developed the training framework and learning objectives across three thematic domains: nursing management, nursing education, and chronic disease prevention with a focus in smoking cessation. The overarching goal was to enhance emerging nurse leaders’ knowledge, attitudes, and competencies in leadership, education, and community health.

The TTT program was designed to accommodate diverse learners by employing modular course content with clearly defined training objectives and learning outcomes for each target group. The modular structure supported a flexible, scalable and ongoing professional development across different healthcare settings (Table 2). The curriculum aimed to: (1) strengthen leadership and management competencies of nurse managers; (2) enhance teaching and pedagogical skills of nurse educators; (3) improve nurses’ capacity in chronic disease prevention and tobacco dependency treatment; (4) establish a provincial network of nurse trainers to support knowledge dissemination and peer mentoring; and (5) develop a scalable Train-the-Trainer model applicable to other provinces in China. These insights directly guided the subsequent phase of curriculum development and implementation.

**Table 2:** Curricula content of the TTT program for advancement in nursing

Course 1: Nursing Management	Course 2: Tobacco Dependency Nursing Intervention and Management	Course 3: Nursing Education
**Week 1 (Guangzhou, China)**	**Week 1 (Guangzhou, China)**	**Week 1 (Guangzhou, China)**
Healthcare reform in China	Public health, health care reform, and chronic disease prevention in China	Theory and practice gap in nursing education
Management principles	The tobacco epidemic worldwide and in Hong Kong/China	- The Clinical Mentor Scheme
Overview roles of manager in healthcare services	Health consequences of active and passive smoking	- The Clinician Educator Initiative
Time management and delegation	Nicotine dependence and addiction theory	- Clinical Problem-based Learning
Effective communication	Types of pharmacological products e.g. Nicotine replacement therapy	Learning styles and strategies
Leadership theories/models	The benefits of smoking cessation	- A deep approach to learning
Team building		- Large class teaching and small group learning
Organizational conflict and conflict management		- Provide positive feedback to students
Staff management		
**Week 2 (Hong Kong)**	**Week 2 (Hong Kong)**	**Week 2 (Hong Kong)**
Trends of healthcare in Hong Kong/internationally	Tobacco control, policy, advocacy, and smoking prevention program in Hong Kong	Philosophy of education
Organizational development in healthcare	The stages of readiness to quit and its application to smoking cessation	Theories of Learning
Leading and managing change	Smoking cessation interventions (behavioral and pharmacological) and treatment plan	Ways in which people learn
Business process re-engineering	How to conduct 5 “A” and 5 “R” interventions	Construction of a syllabus
Stress management	Telephone and group counseling	- Problem-based learning
Quality assurance and its related techniques	Relapse prevention and follow-up	Effective evaluation of classroom and clinical teaching
Risk management in healthcare services	Validation of quitting e.g. Carbon Monoxide monitoring, urine cotinine	- Written examination
Managing health and safety in the workplace	Ex-smoker’s sharing	- Clinical examination
Public accountability and consumerism	Educational visits to hospital- and community-based smoking cessation clinics/centers	- Objective Clinical Structured Assessment/Examination
Manpower planning	International workshops/Conference in tobacco control and smoking cessation	Information technology
Information system for health and management		Observed teachings
Departmental management		Educational visits to hospitals
Planning and managing projects		
Field visits to hospitals		
**Week 3 (Hong Kong)**		
Clinical attachment in hospitals		

#### Development phase – course creation and instructional materials

Based on the established training framework and specific training objectives, three courses were developed: *Nursing Management*, *Tobacco Dependency Nursing Intervention and Management*, and *Nursing Education*. The curricula content and training materials were designed in accordance with the program objectives (Table 2). Instructional materials were adapted from existing best practices and developed in collaboration with experienced nurse educators. A variety of teaching methods were used, including didactic lectures, interactive tutorials, skill-based workshops, field visits, clinical attachment in hospitals, and community healthcare settings to ensure practical relevance. Educational resources included slide decks, case scenarios, checklists, evaluation tools, and teaching guides, tailored to support cascade training upon course completion.

Each course incorporated tailored teaching strategies. The *Nursing Management* course emphasized interactive learning through case discussions, role play, and management simulations, encouraging participants to develop solutions based on their own experiences. Following participant feedback, a one-week clinical attachment in Hong Kong public hospitals was added for later cohorts to provide hands-on learning opportunities. In the *Tobacco Dependency Nursing Intervention and Management* course, participants attended professional conferences and workshops on tobacco control and smoking cessation in Hong Kong. The *Nursing Education* course included classroom observations and institutional visits to deepen participants’ understanding of nursing education systems and the role of nurse educators in Hong Kong.

#### Implementation phase – program delivery

Each module was implemented through a structured two-week format combining classroom instruction, experiential learning, and field exposure. Courses were delivered in both Guangzhou and Hong Kong by experienced faculties including five professors from Nursing Schools all around the world. The first week took place in Guangzhou and focused on foundational content, with local and Hong Kong experts co-facilitating lectures, workshops, and group discussions to ensure contextual relevance. The second week was conducted in Hong Kong, emphasizing advanced knowledge application through case-based learning, clinical attachments, and field visits to hospitals, community health centres, and smoking cessation clinics. To maintain curriculum fidelity and quality, standardized teaching materials and session plans were developed and used by all instructors.

Participants actively engaged in group projects, case presentations, and reflective discussions designed to promote peer learning and leadership development. Real-time feedback sessions were integrated throughout the program to consolidate learning, strengthen confidence, and facilitate the translation of new knowledge and skills into clinical and community practice.

#### Evaluation phase – data collection

To assess the short-term, long-term, and cascading effects of the training intervention, we adopted Kirkpatrick’s Four-Level Training Evaluation Model as the guiding framework for the evaluation phase [25]. Both process and outcome evaluations were conducted at different time points to assess program fidelity, quality and effectiveness. Participants’ satisfaction with the courses, knowledge, attitudes, and practices (KAP) towards the respective course focus, and its individual and systems outputs (e.g. TTT activities) and outcomes were measured.

##### Survey instrument development and validation

The KAP survey tools were adapted from previously validated instruments used in our prior nursing and healthcare training programs. These tools were customized to align with the specific learning objectives of each TTT course. A pilot test was conducted with a small group of nurses to ensure clarity, relevance, and internal consistency. Feedback was used to revise the instruments accordingly.

##### Short-term evaluation

On day 1 of each course, before the first session, all participants were invited to complete the pre-course survey assessing their baseline knowledge and competency in relation to the course contents. In-course evaluation included individual reports and group presentations focusing on the planning, implementation, and evaluation of post-course TTT activities. On the final day of the course, they were invited to complete the post-course survey as well as course evaluation, which measured course satisfaction and self-reported improvements in knowledge, attitudes, and competencies using a 5-point Likert scale.

##### Medium-term evaluation

At 6-month post-course, participants submitted a final report of their cascading activities and completed an online follow-up survey for the overall course evaluation. This phase assessed the initial cascading effect and application of learning, including the number and type of nurses further trained, educational activities conducted, new initiatives launched, and positive changes implemented at participants’ worksites.

##### Long-term evaluation

In 2023, 12 years after TTT program completion, participants were recontacted to assess long-term outcomes through an online survey. The inclusion criteria included: (1) completed the TTT program between 2010 and 2012; (2) Currently employed and working in a relevant field; (3) Had access to electronic devices (e.g., computer, smartphone, or tablet) and could complete the online questionnaire; and (4) Voluntarily participated in the study and provided informed consent. Those who were retired or no longer working in a related position and could not be contacted after three attempts via original communication methods were excluded from the long-term follow-up. This online survey, taking reference from the 6-month post-course survey, examined sustained individual and system changes in nurse management skills, teaching abilities, smoking cessation knowledge and practices, academic impact, and career progression.

##### Cascading impact evaluation

The cascading effect from the TTT program was evaluated by self-reported data in the final reports and 12-year follow-up survey. These included the number of nurses further trained, the scope of knowledge dissemination, and the extent of institutional changes initiated as a result of the participants’ post-training activities.

### Data analysis

Descriptive analysis was used for demographic data, while multivariate analysis of variance (MANOVA) and paired t-tests were employed to analyze pre- and post-program effects. A *p*-value < 0.05 was considered as statistically significant. Cascading effects were assessed through reports of TTT activities and institutional changes.

### Ethical consideration

Ethical approval for conducting both the TTT program (NO: UW: 10–036) and the 12-year follow-up survey (NO: UW 24–131) was obtained from the Institutional Review Board of the University of Hong Kong/Hong Kong Hospital Authority Hong Kong West Cluster. Informed consent was obtained from all participants prior to their involvement in the TTT program and the follow-up survey.

## Results

The TTT program aimed to develop a cohort of nurse trainers with advanced pedagogical, management, and community health skills to strengthen nursing capacity across Guangdong Province. Informed by a needs assessment, we designed targeted training objectives and a structured curriculum. Evaluation of both short- and long-term outcomes showed that the program achieved its goals at individual and institutional levels. Participants reported significant improvements in teaching and leadership competencies, with many of these gains sustained up to 12 years post-training. At the system level, nearly all participating institutions implemented changes in nursing education, management, or clinical practice, demonstrating the program’s cascading impact on workforce development and healthcare quality. Detailed findings are presented below.

### Short- and medium-term outcome evaluation of individual courses

#### Nursing management course

##### Increase in knowledge and competence

**Fig. 1:**
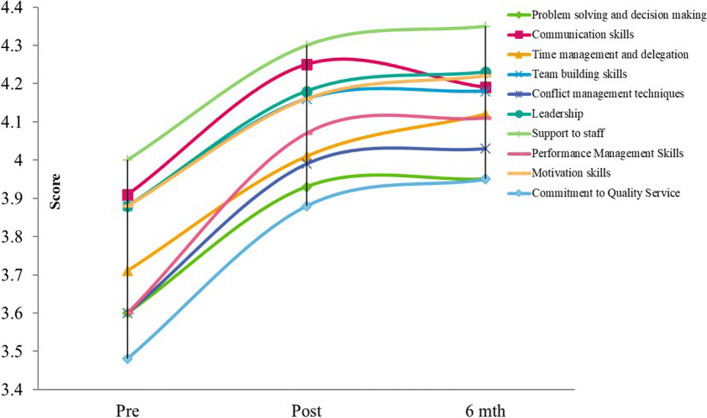
Perceived changes in competence on management ability and skills at pre-, post-course, and 6-month after the course. The items were scored on a scale of 1 (Very incompetent) to 5 (Very competent)

Figure 1 shows the change in competency of participants on management ability and skills, on a scale of 1 to 5 (1 = very incompetent; 5 = very competent). Overall, the scores for all subscales increased significantly immediately post-course and 6-month post-course. The subscale with the highest competency score was “Support to staff” (pre mean = 4.00, post mean = 4.30, 6-month mean = 4.35; *p* < 0.001), while “Commitment to quality service” was the subscale with the lowest competency score (pre mean = 3.48, post mean = 3.88, 6-month mean = 3.95; *p* < 0.001). Further, participants also had an increase in all subscales of management ability and skills immediately after the course. The most improved subscale was “Performance management skills” (mean difference = 0.51, *p* < 0.001). Participants had further increase in most of their management ability and skills 6-month after the course as compared to immediately after, except there was a slight decrease in “Communication skills” (mean difference = −0.06).

##### Change in practice

The course participants reported a change in their management style after attending the training. Almost all of them (98.8%) could effectively perform their duties as a nurse manager and 100% reported changes in management methods. All of them reported improved work in the ward and 86.9% improved their performance in the hospital. Patients were satisfied with the changes and the care they received.

##### Cascading effect of the TTT activities

Ninety participants shared their knowledge with a total of 11,831 nurses. The average number of nurses that each of them further trained was 131. After the cascading activities, a total of 367 institutional changes were made in the 81 institutions, with 78 institutions made at least one institutional change, as follows: 82.7% (67/81) hospitals had implemented new management initiatives to enhance their daily work in the ward; 67.9% (55/81) hospitals set up “risk management system” for quality improvement of patient services; 55.6% (45/81) hospitals implemented the “named nurse” system for providing more holistic and better care to patients; 38.3% (31/81) hospitals introduced new “staff development programs” that they learned from Hong Kong for their own nurses; 28.4% (23/81) hospitals reported that their patient satisfaction improved after they had implemented the new management initiatives.

#### Tobacco dependency nursing intervention and management course

##### Increase in knowledge and competence

**Fig. 2:**
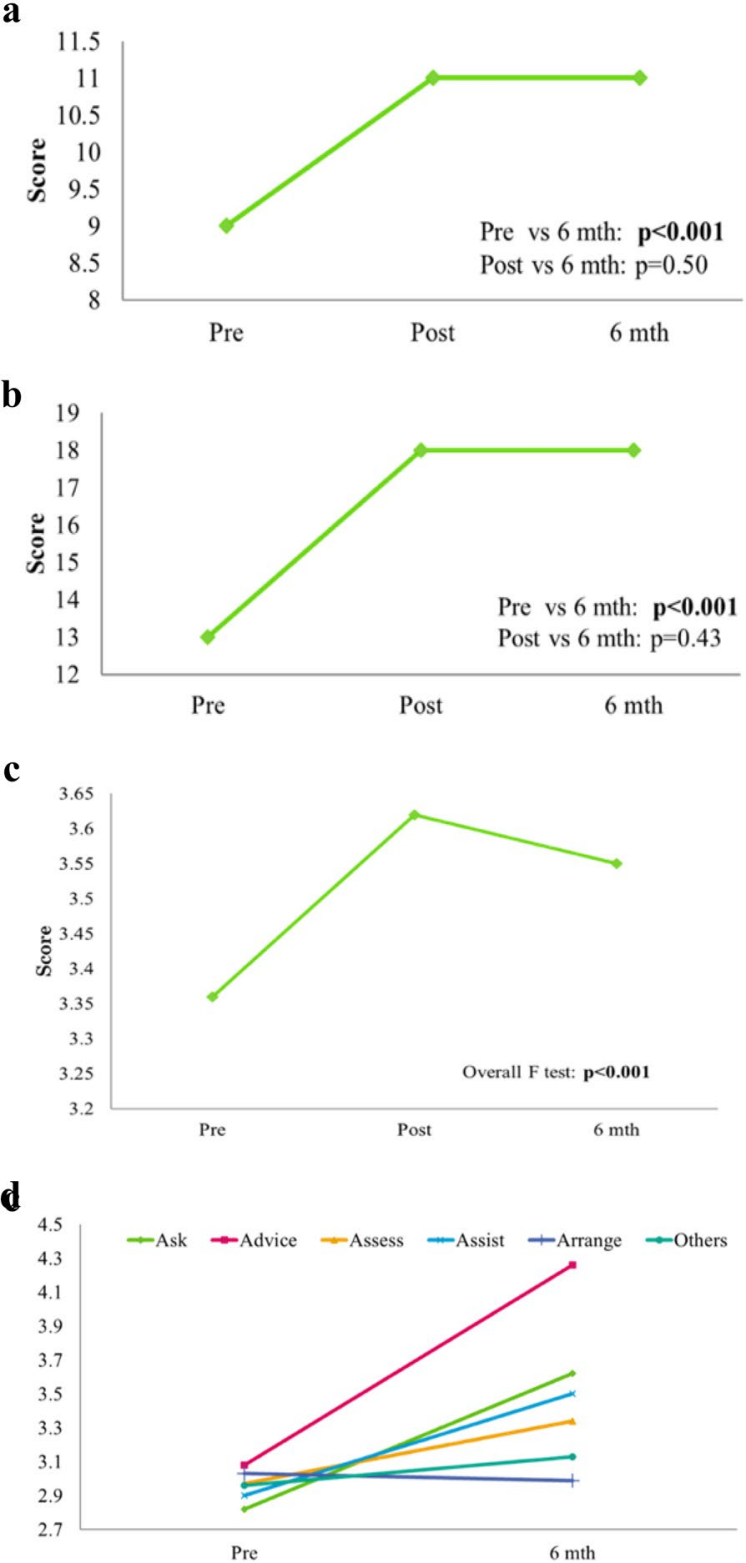
**a** Perceived change in participants’ knowledge on smoking and health at pre-, post-course, and 6-month after the course. **b** Perceived change in participants’ knowledge on the relationship between smoking and diseases at pre-, post-course, and 6-month after the course. **c** Perceived change in participants’ attitudes towards smoking cessation at pre-, post-course and 6-month after the course. The item was scored on a scale of 1 (Strongly disagree) to 4 (Strongly agree). **d** Perceived change in participants’ frequency of performing interventions to help patients stop smoking at pre- and 6-month after the course

Figure 2a and b show the change in participants’ knowledge and competency in performing smoking cessation interventions. Immediate and 6-month post-course evaluations showed that the participants made significant improvements related to health- (median = 9 vs. 11, *p* < 0.001) (Fig. 2a) and disease-related knowledge on smoking (median = 13 vs. 18, *p* < 0.001) (Fig. 2b); had more positive attitudes toward tobacco control and smoking cessation (mean = 3.36 vs. 3.62, *p* < 0.001) (Fig. 2c); and were more competent to perform tobacco dependency treatment (mean = 2.83 vs. 3.21, *p* < 0.001) (Fig. 2d) compared to pre-course. The course had a significant effect on the overall level of competence of participants in performing smoking cessation interventions. Participants practiced smoking cessation counselling more frequently by asking patients about their smoking status (mean = 2.85 vs. 3.65, *p* = 0.001); advising smokers to stop smoking (mean = 3.10 vs. 4.24, *p* < 0.001); assessing their readiness to quit (mean = 2.93 vs. 3.36, *p* = 0.03); and providing smoking cessation assistance (mean = 2.92 vs. 3.50, *p* = 0.001).

##### Change in practice

After the training, the vast majority (96.3%) of them made changes in tobacco control efforts and strategies, 100.0% of them effectively performed the role of nurses/public health staff in tobacco control, 98.1% effectively improved tobacco control work in the wards while 87.0% effectively improved tobacco control work in the hospital.

##### Cascading effect of the TTT activities

Up to six months after the training, 59 participants organized over 100 cascading workshops and further trained 10,621 nurses/public health physicians in Guangdong Province in tobacco control advocacy and smoking cessation. The average number of people that each of them further trained was 180. After the cascading activities, a total of 248 institutional changes were made in 53 institutions, with all institutions made at least one institutional change, as follows: 86.8% (46/53) hospitals/health education institutions actively publicized the smoking cessation messages through different channels, in particular the mass media; 67.9% (36/53) institutions successfully implemented “strategies” to discourage smoking in the work place; 49.1% (26/53) institutions implemented the policy of “smoke free” working environment and 39.6% (21/53) institutions set up an area designated for “smoking” outside the office building; 56.6% (30/53) institutions conducted “tobacco control” training for their staff.

#### Nursing education course

##### Increase in knowledge and competence

**Fig. 3:**
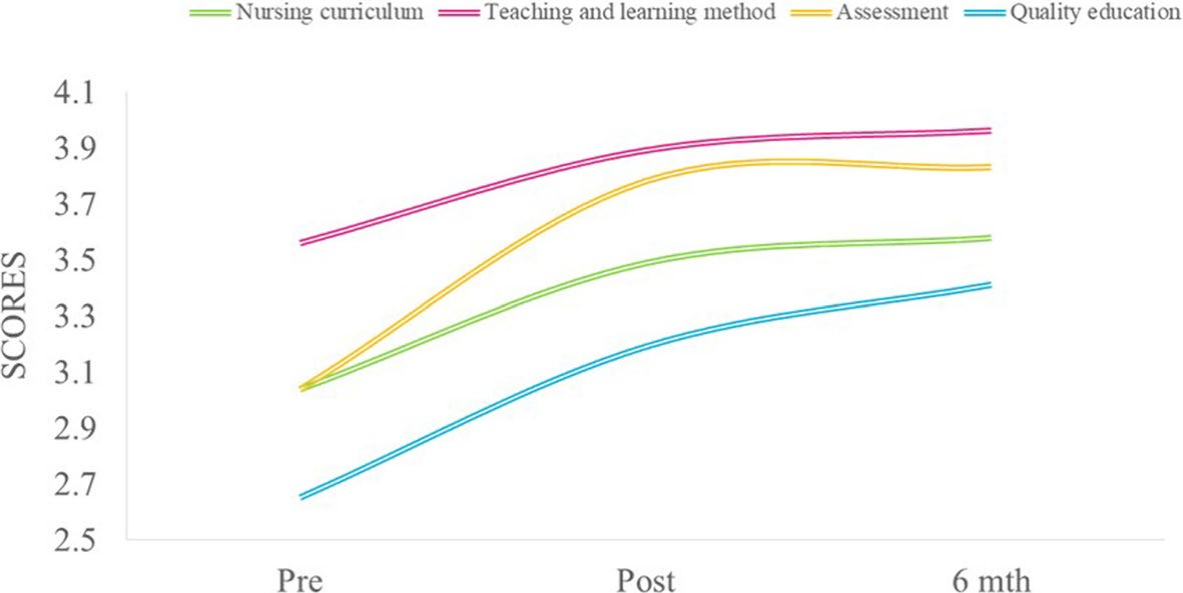
Perceived change in participants’ competency on teaching ability and skills at pre-, post-course and 6-month after the course. The items were scored on a scale of 1 (Very incompetent) to 5 (Very competent)

Figure 3 shows the change in participants’ competency in teaching ability and skills. Overall, significant increases were reported in all four categories of teaching ability and skills (on a scale of 1 = very incompetent to 5 = very competent) immediately after the course compared to pre-course, and they were further improved after six months. The category with the highest competency score was “Teaching and learning methods” (pre mean = 3.56, post mean = 3.89, 6-month mean = 3.96; *p* = 0.001).

##### Change in practice

The course participants applied different teaching and assessment methods in their daily work and enriched the quality of their teaching as well as enhanced their role as a nurse educator. They made changes in nursing education work (100.0%), effectively performed the role of nurse educator (100.0%), effectively improved teaching quality of the university schools of nursing (80.0%), and effectively improved the overall education performance in the university schools of nursing (96.0%).

##### Cascading effect of the TTT activities

The total number of nurse teachers with whom the 30 participants shared their newly acquired knowledge was 2,262. The average number of nurse teachers that each of them further trained was 75. After the cascading activities, a total of 55 institutional changes were made in 18 institutions, with all institutions made at least one institutional change, as follows: 61.1% (11/18) nursing schools had applied the “deep approach of learning” mode in teaching and this was well received by most students; 55.6% (10/18) nursing schools adopted new methods for student assessment; among which 4 schools tried out the “Objective Structure Clinical Examination (OSCE)” in the final examination; 50.0% (9/18) nursing schools had piloted “clinical mentorship” and it was planned to launch this scheme in the GD Province after the pilot because feedback from the students was very favourable including enhanced learning; 44.4% (8/18) schools had used positive feedback techniques during teaching and was well received by the students; and 38.9% (7/18) schools adopted problem-based learning in their curriculum.

#### The 12-year follow up evaluation

In 2023, all the 179 course participants were recontacted to join the 12-year follow-up survey, 101 (56.4%) responded and completed the online survey. It included 54 participants from the Nursing Management courses, 21 from Tobacco Dependency Nursing Intervention and Management courses, and 26 from the Nursing Education course. The vast majority (94.1%; n = 95) were female. The participants had a mean age of 48.9 years (range: 38 to 60 years) and an average work experience of 28.3 years (range: 16 to 43 years). Regarding educational background, 81 participants held a bachelor’s degree (80.2%), while 20 held master’s or doctoral degrees (19.8%). Additionally, 92 participants held advanced professional titles (91.1%).

##### Changes in knowledge, competence, and practice

Comparing immediately post-training and at the 6-month follow-up with 12 years post-course, significant improvements were observed among the 54 Nursing Management course participants in problem-solving, decision-making, communication skills, teamwork, conflict management, management skills, and service quality commitment (Table 3). Similarly, the 26 participants in Nursing Education course demonstrated significant enhancements in teaching abilities, including nursing curriculum, teaching methods, teaching assessment, and delivery of high-quality nursing education. Nevertheless, the 21 participants in Tobacco Dependency Nursing Intervention and Management courses, many has changed jobs, hence exhibited a decrease in self-reported smoking cessation intervention practices.

**Table 3:** Perceived changes in participants’ knowledge, competence, and practice at pre-, post-course, 6-month, and 12 years after the course

	Before TTT program Mean ± SD	Immediately post-TTT Mean ± SD	6-month post-TTT Mean ± SD	12 years post-TTT Mean ± SD	Immediately vs. 12 years post-TTT *p*-value	6 months vs. 12 years post TTT *p*-value
Nursing management skills	*n* = 90	*n* = 90	*n* = 90	*n* = 54		
1) Solve problems and make decisions	3.48 ± 0.58	3.83 ± 0.52	3.92 ± 0.48	4.17 ± 0.61	< 0.001	0.01
2) Communication skills	3.86 ± 0.51	4.24 ± 0.50	4.16 ± 0.45	4.31 ± 0.41	0.39	0.05
3) Time management and delegation	3.70 ± 0.55	3.98 ± 0.52	4.04 ± 0.56	4.20 ± 0.45	0.01	0.08
4) Group cooperation	3.90 ± 0.48	4.12 ± 0.47	4.13 ± 0.43	4.35 ± 0.48	0.01	0.01
5) Management skills when arguing	3.54 ± 0.58	3.94 ± 0.55	3.97 ± 0.49	4.14 ± 0.51	0.03	0.05
6) Leadership	3.89 ± 0.51	4.17 ± 0.46	4.21 ± 0.53	4.32 ± 0.48	0.06	0.21
7) Support for caregivers	4.07 ± 0.49	4.30 ± 0.53	4.37 ± 0.57	4.42 ± 0.59	0.21	0.62
8) Performance of management skills	3.49 ± 0.64	3.99 ± 0.57	4.07 ± 0.52	4.30 ± 0.60	0.002	0.02
9) Motivational skills	3.94 ± 0.55	4.16 ± 0.42	4.22 ± 0.58	4.36 ± 0.51	0.01	0.15
10) Commitment to service quality	3.43 ± 0.60	3.82 ± 0.58	3.90 ± 0.52	4.20 ± 0.48	< 0.001	< 0.001
Nursing teaching ability	*n* = 30	*n* = 30	*n* = 28	*n* = 26		
1) Nursing courses	3.03 ± 0.96	3.60 ± 0.56	3.61 ± 0.48	4.03 ± 0.46	0.003	0.002
2) Teaching methods	3.47 ± 0.63	3.80 ± 0.55	4.00 ± 0.43	4.38 ± 0.52	< 0.001	0.01
3) Teaching evaluation	2.90 ± 0.84	3.60 ± 0.67	3.80 ± 0.57	4.15 ± 0.37	< 0.001	0.01
4) High-quality nursing education	2.53 ± 0.86	3.10 ± 0.80	3.41 ± 0.58	3.92 ± 0.77	< 0.001	0.01
Tobacco control management capacity	*n* = 59	*n* = 59	*n* = 56	*n* = 21		
1) Assess the history and current status of smoking in smokers	2.80 ± 0.71	3.37 ± 0.49	3.27 ± 0.45	3.00 ± 0.45	0.003	0.02
2) Educate smokers on how to quit smoking	2.86 ± 0.61	3.27 ± 0.45	3.34 ± 0.48	2.95 ± 0.38	0.003	0.001
3) Health education on smoking cessation	3.10 ± 0.55	3.40 ± 0.50	3.46 ± 0.50	3.10 ± 0.44	0.02	0.01
4) Understand smokers' beliefs/perceptions about smoking and health	2.96 ± 0.57	3.37 ± 0.49	3.30 ± 0.54	3.10 ± 0.43	0.03	0.13
5) Correct smokers' negative attitudes about quitting	2.81 ± 0.56	3.31 ± 0.47	3.20 ± 0.48	3.00 ± 0.45	0.01	0.10
6) Assist smokers in developing a quit plan, including setting a quit date	2.74 ± 0.71	3.43 ± 0.50	3.16 ± 0.37	2.90 ± 0.54	< 0.001	0.02
7) Discuss different ways to quit smoking	2.79 ± 0.66	3.41 ± 0.50	3.20 ± 0.40	2.76 ± 0.63	< 0.001	< 0.001
8) Give advice on nicotine replacement therapy	2.62 ± 0.69	3.18 ± 0.43	3.04 ± 0.47	2.43 ± 0.68	< 0.001	< 0.001
9) Use small flyers or other written materials to help smokers quit	3.07 ± 0.53	3.63 ± 0.49	3.54 ± 0.54	3.10 ± 0.44	< 0.001	0.001
10) Use a carbon monoxide monitor to measure the carbon monoxide content of the smoker's exhaled breath	2.15 ± 0.82	3.05 ± 0.61	2.65 ± 0.83	2.10 ± 0.63	< 0.001	0.01
11) Communicate with the smoker's family	3.00 ± 0.54	3.36 ± 0.52	3.25 ± 0.48	3.05 ± 0.50	0.02	0.11
12)Tips to prevent relapse after quitting smoking	2.74 ± 0.76	3.31 ± 0.50	3.23 ± 0.43	2.90 ± 0.54	0.002	0.01
13) Monitor smoking/secondhand smoke in the community	2.49 ± 0.76	2.87 ± 0.59	2.74 ± 0.71	2.24 ± 0.77	< 0.001	0.01
14) Collect information on tobacco control and smoking cessation	3.09 ± 0.54	3.41 ± 0.50	3.35 ± 0.52	3.00 ± 0.63	0.004	0.02
15) Preparation and production of educational materials on smoking and health/smoking cessation	3.02 ± 0.61	3.39 ± 0.53	3.25 ± 0.61	2.71 ± 0.64	< 0.001	0.001
16) Train other community health care workers in smoking cessation interventions	2.87 ± 0.58	3.36 ± 0.48	3.32 ± 0.47	2.67 ± 0.66	< 0.001	< 0.001

Results were presented as mean ± sd; significance was tested by paired t-test

##### Cascading effect of the TTT activities

Over the past 12 years following the TTT program, participants in the Nursing Management courses shared knowledge with approximately 40,954 colleagues, averaging 758 colleagues per participant. Participants in Tobacco Dependency Nursing Intervention and Management courses shared knowledge with approximately 2,946 colleagues, while those in the Nursing Education course shared with approximately 5,485 colleagues, with averages of 113 and 261 colleagues per participant, respectively. These findings suggested a sustained cascading effect in general, as evidenced by the increasing number of individuals to whom participants disseminated knowledge or provided further training compared to reports at the 6-month post-TTT period.

## Discussion

This paper discussed a special designed TTT curricula model for emerging nurse leaders in advancing nursing in South China’s Greater Bay Area, with a built-in rigorous reporting mechanism and a longitudinal evaluation 12 years post-TTT. The TTT program demonstrated success in meeting the program goal of creating a critical mass of nurse trainers who transferred their newly acquired knowledge and skills to other nurses in the Guangdong Province. The course participants shared their knowledge and skills acquired from the courses with about 25,000 nurses in Guangdong province within 6-month post-TTT. The specific program objectives—enhancing the knowledge, skills, and competencies of emerging nurse leaders—were achieved at both individual and system levels. At the individual level, the course participants showed improvement in their knowledge, competencies, and capability immediately after and six months post-courses with positive changes mostly sustained 12 years after the program. These outcomes directly reflect the success of Objectives 1, 2, and 3, highlighting the program’s effectiveness in strengthening teaching and leadership skills over time. At the institutional level, 134 of the 136 (98.5%) participating institutions made at least one institutional change, with a total of 670 institutional changes improving work in nursing management, tobacco control and smoking cessation, and nurse education activities, aligning with the program’s goal of broader systemic change and illustrating its cascading impact.

The TTT educational approach has proven feasible, practical, and effective across diverse settings, enhancing nurses’ knowledge, skills, and self-efficacy in areas such as cancer care [26–28], chronic diseases management [29–32], infectious diseases control [33–35], hospice and palliative care [36], in emergency department [37], and primary care clinics [38]. Our study aligns with previous evidence showing the feasibility and effectiveness of the TTT approach in healthcare education.

Notably, our TTT program proved to be highly scalable. Within six months after the program, participants had trasferred their knowledge to about 25,000 secondary trainees (nurses and health educators, representing 20% of the nursing workforce) in the Province. This far exceeds the reach of comparable TTT programs in other regions or fields, for instance those for chronic fatigue syndrome (reaching 2,064 individuals) [29], dementia (reaching 3,276 individuals) [32], and alcohol abuse (reaching 2066 trainees) [31]. Our TTT program also stands out for its inclusion of a 12-year follow-up, rare in training evaluation literature, demonstrating sustained knowledge application.

Furthermore, the “1 + 1” training model adopted by our TTT program in which the training courses took place in both Guangzhou and Hong Kong, allowed efficient utilization of resources, localized adaptation of the teaching materials, and cultural exchange. For example, nurse participants learnt smoking cessation strategies in Hong Kong (e.g. telephone counseling, motivational interviewing, nicotine gum or patch) and participated in classroom-based learning modelled after the Hong Kong nursing curriculum. This cross-board enriched learning experience fostered professional networks and created a shared vision for cultivating future nursing leadership.

The TTT program also aligns with broader health system goals, particularly the Healthy China 2030 and Healthy China Action Plan by the National Health Commission of China, which emphasize optimizing healthcare services, especially primary care, as amid epidemiological transition, sociodemographic shift, and economic pressures [19, 20, 39]. With China facing a projected shortfall of almost 2 million nurses by 2030 [40], innovative training models like TTT are critical. Several pilot programs have begun training nurses as primary care providers in urban community centres [41], yet questions such as “What should the curriculum include?” and “How well will Nurse Practitioners perform?” remain. Our TTT program, demonstrated a new prototype for scalable, competency-based training initiative and curriculum that not only enhances individual capacity but also drives institutional change.

The Healthy China 2030 Action Plan highlighted the importance of tobacco control so as to decrease adult smoking prevalence to 20% by 2030. This commitment is an ambitious goal for tobacco control given that China has the world’s largest number of manufacturers of cigarettes and that overall adult smoking prevalence has been decreasing slowly [42, 43]. The Tobacco Dependency Nursing Intervention and Management course in our program had successfully enhanced the knowledge and skills among individual nurses and public health professionals. However, our long-term evaluation revealed a decline in sustained impact, unlike the consistent improvements seen in the other two courses. The decline likely resulted from staff turnover and limited institutional support for tobacco control and smoking cessation, which are often not revenue-generating. This discrepancy is notably influenced by the job roles of the 21 participants. Specifically, the 12 nurses actively involved in direct patient care within oncology, respiratory, and pulmonary departments, continued to assist a significant number of smokers in quitting—up to 50 each. In contrast, the nine physicians, primarily in management roles, had fewer opportunities to apply what they learnt, highlighting the importance of an enabling environment to facilitate sustained practice.

This underscores the critical need for regular reinforcement, institutional support, and policy alignment to maintain the progress. Financial constraints and the marginalization of tobacco control and smoking cessation further hinder the sustainability of such practice. A robust public health policy and financial support mechanisms are essential to ensure that smoking cessation becomes a routine part of clinical practice. Despite these challenges, our nurse-led smoking cessation program remains a promising model and, if scaled and integrated effectively, could significantly contribute to achieving the national tobacco control targets.

Looking ahead, we could first leverage the Greater Bay Area as an “entry point” to China. With proximity between Guangdong and Hong Kong, major cities, such as Shenzhen, Zhuhai, and Zhongshan, could host training hubs that replicate and adapt our model [44]. A comprehensive needs assessment would be useful to tailor training curricula to address the regional and global healthcare megatrends and challenges. With technological advancement, e-learning via digital platform, artificial intelligence-assisted simulations, gamified learning modules, and chatbots could also be incorporated into TTT programs to ensure engagement and accessibility [45].

## Conclusions

In conclusion, our TTT program provides an innovative, evidence-based model for nursing education and capacity building, fostering institutional change and responding to national healthcare priorities. Unlike traditional models that often lack scalability, long-term evaluation, or cross-sector collaboration, this program offers a sustainable framework for health workforce development, with potential applications across China and globally.

## Data Availability

The datasets, analysis, and questionnaires that were used will be made available from the corresponding author upon reasonable request.

## Abbreviations

NCDs: Non-communicable diseases
TTT: Train-the-Trainer
GD: Guangdong Province
HKU: The University of Hong Kong
KAP: Knowledge, attitudes, and practices
